# Rapid increase in immune surveillance and expression of NKT and γδT cell activation markers after consuming a nutraceutical supplement containing *Aloe vera* gel, extracts of *Poria cocos* and rosemary. A randomized placebo-controlled cross-over trial

**DOI:** 10.1371/journal.pone.0291254

**Published:** 2023-09-12

**Authors:** Liu Yu, Sage McGarry, Dina Cruickshank, Gitte S. Jensen

**Affiliations:** 1 NIS Labs, Port Dover, Ontario, Canada; 2 NIS Labs, Klamath Falls, Oregon, United States of America; University of Milan, ITALY

## Abstract

**Goal:**

To evaluate the acute impact of a nutraceutical blend on immune surveillance.

**Study design:**

A randomized, double-blind, placebo-controlled, cross-over trial was conducted in 11 healthy subjects. Blood samples were taken immediately before and at 1, 2, and 3 hours after consuming placebo or 500 mg of UP360, which is a blend of botanicals from *Aloe vera*, *Poria cocos*, and rosemary (APR extract). Immunophenotyping and flow cytometry quantified numbers of monocytes, NK cells, NKT cells, CD8+ cytotoxic T cells, γδT cells, and total T cells, and expression of CD25 and CD69 activation markers. Plasma was tested for cytokines, chemokines, growth factors, and enzymatic activity of superoxide dismutase and catalase.

**Results:**

Compared to the placebo, consumption of APR extract triggered rapid increases in chemokine levels starting at 1 hour, including IP-10 (P<0.05) and MCP-1 (P<0.1), which peaked at 2 hours (P<0.01) and 3 hours (P<0.05), respectively. The stem cell-mobilizing growth factor G-CSF increased at 2 hours (P<0.05). Increased immune surveillance involved a transient effect for monocytes at 1 hour, followed by NKT cells, CD8+ cytotoxic T cells, and γδT cells at 2–3 hours. Increased immune cell alertness was seen at 1 hour by increased CD25 expression on monocytes (P<0.01), NKT cells (P<0.01), and T cells (P<0.05). NKT cells showed upregulation of CD69 at 2 hours (P<0.01). Increased enzymatic activity was seen at 2 hours for the antioxidant enzymes superoxide dismutase (P<0.05) and catalase (P<0.01).

**Conclusion:**

Consumption of APR extract triggered acute changes to chemokine levels. In addition, immune alertness was increased via the expression of activation markers on multiple types of innate immune cells, followed by increased immune surveillance and antioxidant protection. This suggests a beneficial enhancement of natural immune surveillance, likely via a combination of gut-mediated cytokine release and vagus nerve communication, in combination with cellular protection from oxidative stress.

## Introduction

Immune surveillance is a process where immune cells monitor all tissues in the body, on the alert for pathogens and premalignant and malignant cells. During this process, our immune cells travel throughout the body in a resting state, involving cell migration from the blood circulation to the tissue and returning to the blood circulation via the lymphatic system.

Unique anatomical areas are smaller post-capillary veins in which the immune cells travel close to the vessel walls and recognize signals from chemokines secreted from tissue and the endothelial vessel walls [1], directed by nerve endings in the blood vessel walls [2]. These venous areas are ideal for chemotactic adhesion and extravasation of immune cells [3].

As part of the process of migrating from the blood circulation across the endothelial barrier into tissue, the immune cells sequentially engage a series of different types of adhesion molecules, including selectins and integrins, that act as both adhesive and signaling receptors [4–7]. As the circulating cells come to a stop on the endothelium and migrate between adjacent endothelial cells and through the underlying extracellular matrix, this process initiates changes to the alertness of the immune cells from the resting state in the blood circulation [4–6].

The immune system undergoes circadian cycles [8] affecting immune communication and surveillance [9], where neuroendocrine signals bring about changes in immune surveillance in accordance with the time of day and night. Cryptochrome, a molecular clock protein and photoreceptor, assists in the coordination of pro-inflammatory cytokines which are upregulated in the morning as rest ends [10]. Cryptochrome also upregulates glucocorticoids, epinephrine, and norepinephrine, which directly influences the activity of immune cells [11]. Circadian rhythms influence immune cell trafficking between blood, tissue, and lymphatic vessels [12]. Natural killer cell activity increases during the morning and tapers off as the day continues [13]. B-cell and T-cell functions also vary with circadian rhythms wherein the time of day can significantly impact the effectiveness of immune responses [14, 15]. Immune cells also have inherent clocks that regulate immune surveillance, with increased accumulation in lymphatic tissue at night, and increased trafficking during the day [16].

Biological communication networks require a bidirectional communication from a variety of signals to function properly, and disruptions or noise in those signals can inhibit the ability to mount an appropriate immune response [17]. Pattern recognition receptors on antigen-presenting cells such as monocytes and dendritic cells detect potential threats and initiate pro-inflammatory responses [18]. Natural killer cells are also activated via these pathways [19].

This communication is disrupted by stress, disease, and environmental factors [20], where homeostasis is disrupted by psychological, physiological, or environmental threats, and the body responds with inflammatory reactions [21]. The hypothalamic-pituitary-adrenal axis plays a major role in this stress response [22], where stress induces a cascade of hormones including corticosteroids [23], interacting with cellular receptors involved in a multitude of physiological processes, including immunity [24]. Under normal un-stressed conditions, glucocorticoids suppress both innate and adaptive immunity to restore homeostasis [25–28], and the anti-inflammatory impact of glucocorticoids prevents tissue and nerve damage that can result from widespread inflammation [29]. However, when stress becomes chronic, the adaptive actions of glucocorticoids are reduced, and prolonged glucocorticoid exposure can result in glucocorticoid resistance [30], observed in several chronic diseases including rheumatoid arthritis, inflammatory bowel disease, and systemic lupus [31–34]. Chronic stress-mediated inflammation influences immunity by disrupting communication and preventing appropriate responses.

In this process of concerted communication, the gut mucosal barrier is a pivotal area for our immune system to receive and interpret signals and communicate to the rest of the body. Macrophages and dendritic cells are embedded in the mucosal surface, and the dendritic cells extend tentacles directly into the gut lumen, sampling antigens from the gut microbiome and ingested material, including foods and pathogens present in the food [35]. Inside the mucosal tissue, cellular communication includes NK cells and T cells present in lamina propria and Peyer’s Patches. Gut-to-brain communication via the vagus nerve, followed by central nerve system communication to the periphery, is a rapid process, and likely involved in regulating immune surveillance [36].

Consumption of nutraceutical products can support the natural processes of immune surveillance which represents a unique opportunity for the use of botanical extracts in preventive health. Previous research has shown that consuming certain botanical and fungal extracts can induce mild but significant changes to immune surveillance and activation status within hours [37, 38].

UP360, the blend tested in this study, contains two botanical ingredients and one medicinal mushroom extract. The ingredients are extracts from *Aloe vera*, *Poria cocos* mushroom, and *Rosmarinus officinalis* (rosemary) (**Table 1**) and will be referred to as the APR blend.

**Table 1:** Ingredients in consumable test product UP360.

Latin name	Common name	Part	% blending ratio
***Aloe vera***	Aloe	Aqueous/enzymatic concentrate of leaf gel	30%
***Poria cocos***	Fu Ling	Aqueous/ethanolic extract of sclerotium	60%
***Rosmarinus officinalis***	Rosemary	Aqueous/ethanolic leaf extract	10%

*Aloe* has been used historically across many cultures for treatment of gastrointestinal disorders, microbial infections, and inflammatory conditions [39]. *Aloe* polysaccharides constitute a significant portion of the plant, notably acemannan, a polymer of β-(1–4)-acetylated mannose, that contributes to antiviral and antitumoral activity, while promoting cellular and humoral immunity [40–43]. Smaller polysaccharides such as alprogen contribute to immune modulating effects, specifically antiallergic effects [44, 45]. In rabbits, *Aloe* consumption leads to an increase in T lymphocytes [46], and in a mouse model *Aloe* stimulated splenic cytokine production [47].

Mushrooms have been used in traditional medicine in Europe and Asia. *Poria cocos* is a fungus in the family polyporaceae known for its antidepressant and immune modulating properties [48–50]. The *Poria* immunomodulatory protein (PCP) is one of the known bioactive compounds in the mushroom, with a broad spectrum of biological properties including anti-tumor, immunomodulation, anti-inflammatory, and anti-aging effects [51]. PCP activates macrophages via the toll-like receptor 4 (TLR-4) by upregulating Toll/interleukin-1 receptor/resistance protein (TIR) domain containing adaptors which are early components of the TLR signaling pathway [52]. β-glucan is an insoluble and major component of chitin in *Poria* [53, 54], and acts as a ligand for TLR-2 [55] and TLR-4 receptor systems [52, 56]. Dectin-1 and TLR-4 receptor systems are expressed on macrophages and dendritic cells, including those that reside in the gut mucosa [57].

Rosemary is an herb that has been used in folk medicine in Europe, Egypt, China, India, and Mesopotamia dating back two thousand years [58]. An active component is the water-soluble phenolic compound rosmarinic acid, which has antioxidant and anti-inflammatory effects [59]. Another active component in rosemary, carnosic acid, also demonstrates anti-inflammatory properties, attenuating the expression of tumor necrosis factor-alpha (TNF-α), and the downstream pathways associated with proinflammatory responses [60].

The three ingredients in the APR blend elicit immune modulating effects through different mechanisms, targeting different aspects of the immune system and the regulation of inflammation. They have the potential to work in synergy to create a gestalt impact on immune surveillance and activation. *Aloe* and *Poria* affect different aspects of antigen presentation [40, 49]. *Aloe* and Rosemary both impact antioxidant activity and introduce free radical chain terminators, while also increasing the activity of endogenous antioxidant enzymes [40, 41, 43, 59].

Previous research on the APR blend has shown protective effects in a mouse model of LPS-induced acute lung injury, associated with down-regulation of the pro-inflammatory cytokines TNF-α, Interleukin-1 beta (IL-1β), and Interleukin-6 (IL-6) [S1 File]. The APR blend also showed protection from immunosenescence in mice, including increased superoxide dismutase activity and levels of Nrf2 protein [S1 Data].

For this clinical study, healthy adults were tested following an established placebo-controlled, randomized, double-blinded, cross-over study design. The study focused on changes to immune cell communication through cytokines, chemokines, growth factors, immune surveillance, and changes to immune cell alertness through the expression of activation markers, taking each participant’s circadian changes into account [37, 38, 61–64].

## Materials and methods

### Study design

**Fig 1:**
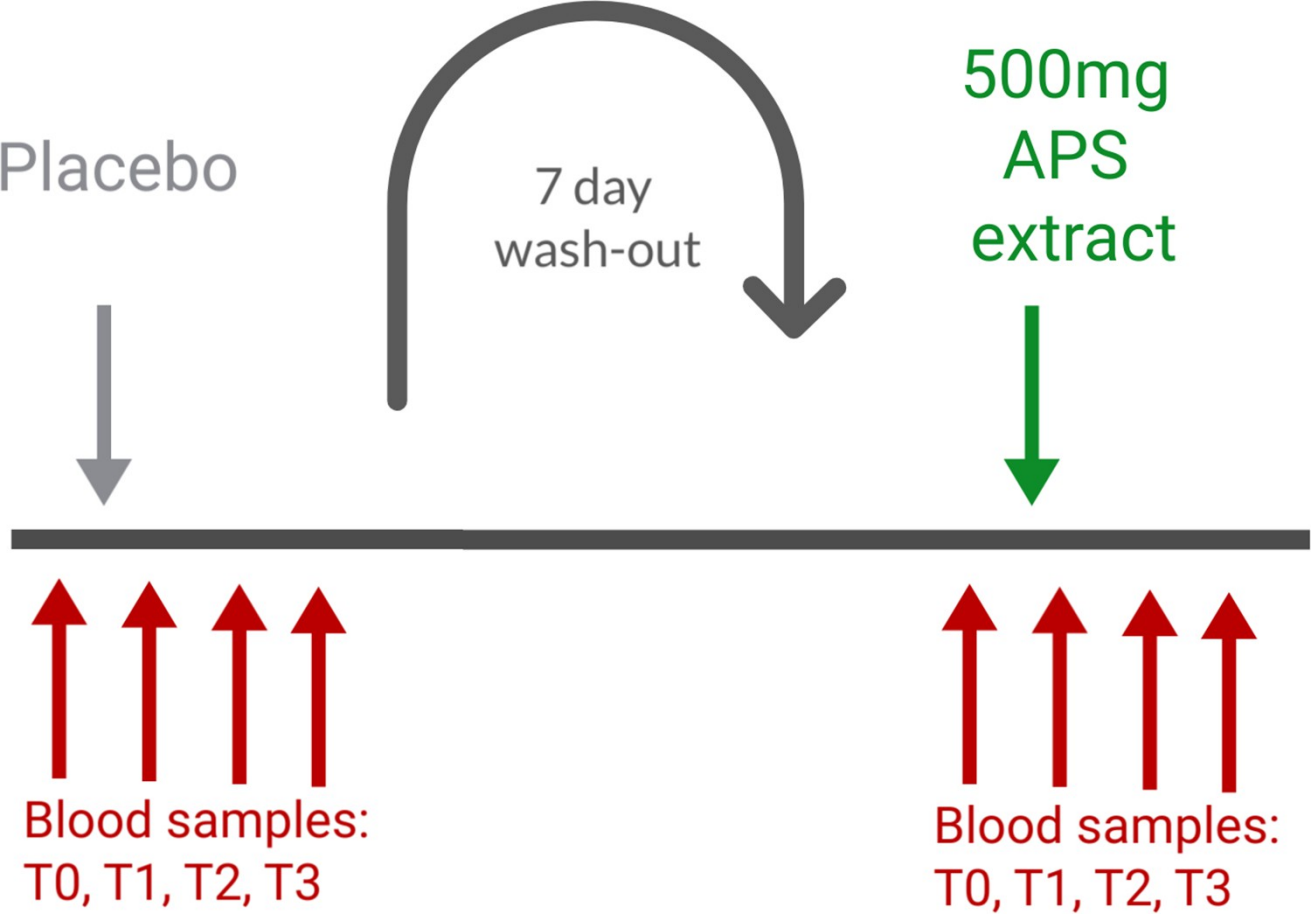
Diagram showing the involvement of each participant. Study participants were tested on two different clinic days. The sequence of test products shown here is an example only, since the sequence was randomized.

**Fig 2:**
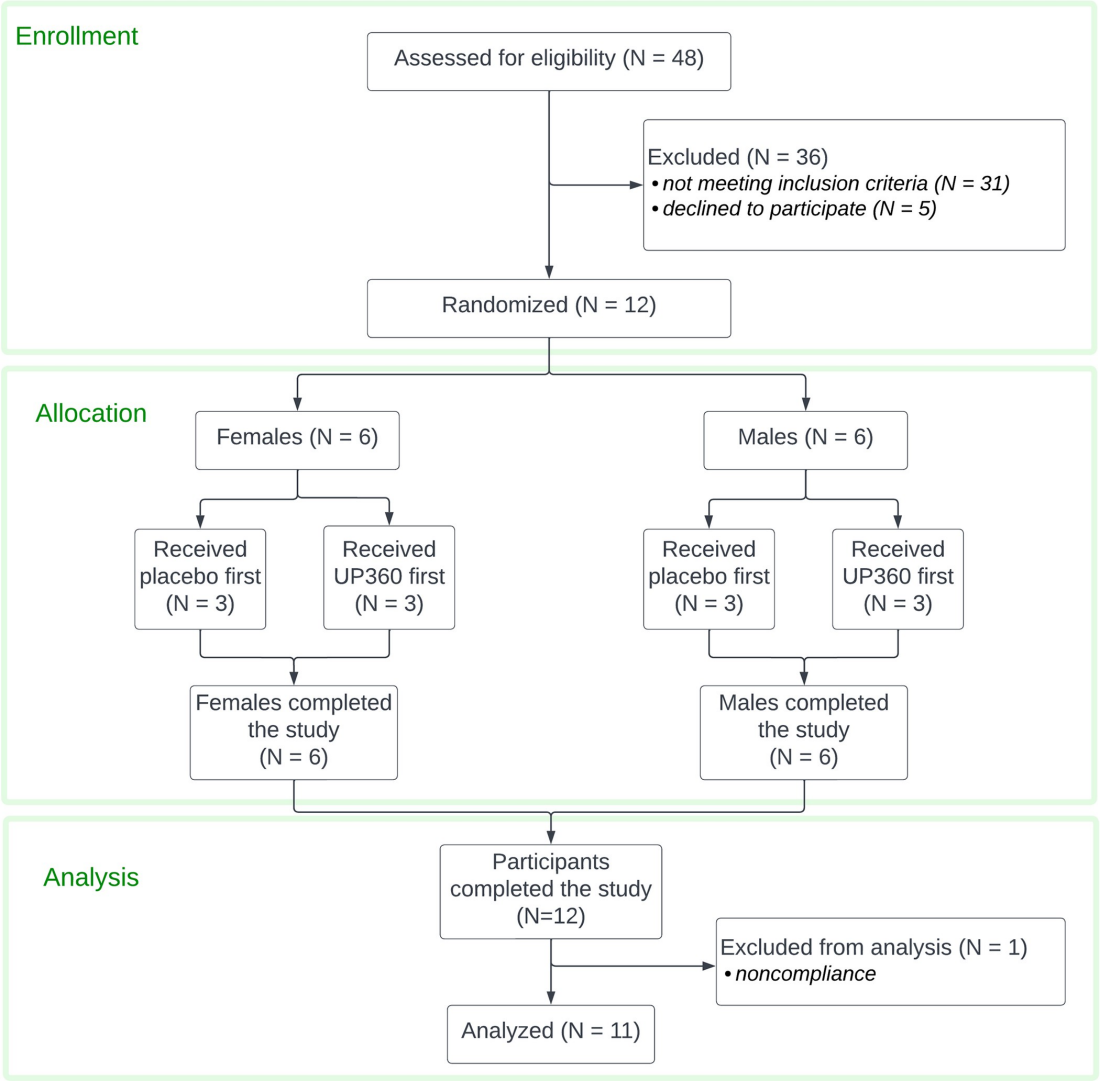
CONSORT flow chart for the study.

A randomized, double-blind, placebo-controlled, cross-over study design was used for this clinical study (clinical trial registration ISRCTN14647763), which was conducted in accordance with the Declaration of Helsinki, and approved by the Argus Independent Review Board, Tucson AZ, USA. The sample size was determined based on previous studies on acute effects of nutraceuticals containing similar doses of beta-glucans and other immune-activating botanical and fungal polysaccharides. Based on the expected effects on innate immune cell types, we aimed for a minimum of 10 people completing the study participation [37, 38]. The study was carried out during September–November 2021 through NIS Labs in southern Oregon, USA, where people live and work at an elevation of 1,200–1,500 meters above sea level. The authors had access to information that identified individual participants during and after data collection. Subjects from the database at the study site, representing people who have previously indicated interest in participating in clinical studies, were approached, and if interested in being considered for the present study, were invited for screening. Forty-eight people went through the screening interview and 17 qualified for participation in this study. Twelve people were enrolled in the study after signing written informed consent, as approved by the institutional review board of Argus IRB Inc. and completed study participation (**Fig 1**). The 12 enrolled participants received the active product and placebo 1 week apart in randomized order. One of the 12 participants who completed the study was excluded from data analysis due to non-compliance (vaccination within 4 weeks of study). As a result, data from 11 participants were included in per-protocol analysis and the data presented here (**Fig 2**).

The screening involved an interview to document age, body mass index (BMI), medical/surgical history, diet and lifestyle, current health status, medication, and use of nutritional supplements. The following inclusion criteria were applied: Healthy adult people of either gender, age 18–75 years (inclusive), body mass index (BMI) between 18.0 and 34.9 kg/m^2^ (inclusive), veins easily accessible for the multiple blood draws, and willing to comply with the study requirements: Maintaining a consistent diet and lifestyle routine throughout the study, consistent habit of bland breakfasts on days of clinic visits, abstaining from exercising and nutritional supplements on the morning of a study visit, abstaining from use of coffee, tea, and soft drinks for at least one hour prior to a clinic visit, and abstaining from music, candy, gum, computer/cell phone use, during clinic visits.

The following exclusion criteria were used: Previous major gastrointestinal surgery (absorption of test product may be altered) (minor surgery not a problem, including previous removal of appendix and gall bladder); taking anti-inflammatory medications on a daily basis; currently in intensive athletic training (such as marathon runners); cancer during past 12 months; chemotherapy during past 12 months; currently treated with immune suppressant medication; diagnosed with autoimmune disorders e.g. systemic lupus erythematosus, hemolytic anemia; donation of blood during the study or within the 4 weeks prior to study start; received a cortisone shot within past 12 weeks; immunization during last month; currently taking antipsychotic hypnotic, or anti-depressant prescription medication; ongoing acute infections (including teeth, sinus, ear, etc.); participation in another clinical trial study during this trial, involving an investigational product or lifestyle change; an unusual sleep routine (examples: working graveyard shift, irregular routine with frequent late nights, studying, partying); unwilling to maintain a constant intake of supplements over the duration of the study; anxiety about having blood drawn; pregnant, nursing, or trying to become pregnant; known food allergies related to ingredients in active test product or placebo. If subjects met the inclusion and exclusion criteria, they were informed that they qualified, and scheduled for clinic visits where they were enrolled into the study upon providing written informed consent. Randomization was performed by assigning the first female study participant to consume product code A on the first clinic visit and consume product code C for her second clinic visit. The sequence alternated for each subsequent female person enrolled. The process was the same for male participants with the first male participant consuming product C on the first clinic visit. The principal investigator generated the random allocation sequence without knowing which participant would be assigned to which sequence. Clinic staff enrolled participants and the scheduling determined the allocation to sequence. All clinic staff were blinded, and blinding was performed by the study sponsor.

Study participants were scheduled for two clinic visits 1 week apart. For each participant, the visits were scheduled at the same time of the day during the morning hours of 7–11 am to minimize the effect of circadian fluctuations [8–11]. One of the two clinic visits involved consuming a placebo and served as a control for the circadian variations in cytokine levels and immune surveillance for each participant. Since there is a well-documented interference from exercise [65] and stress [66–68] with the release versus homing of lymphocytes, the study environment was managed to minimize any physical and mental stress during each visit. Upon arrival to a clinic visit, participants completed a questionnaire to help monitor exceptional circumstances that may have an effect on the stress level of that person on that day. Predetermined criteria for re-scheduling a visit included sleep deprivation and acute anxiety. After completing the questionnaire, volunteers were instructed to remain calm and inactive for 4 hours, comfortably seated in a chair in a private room. After the first hour, the baseline blood sample was drawn. Immediately after the baseline sample was drawn, an encapsulated test product was provided with water and consumed in the presence of the clinic staff. Blood samples were drawn at 1, 2, and 3 hours after consumption of the test product or placebo. For each blood draw, 6 mL of blood was drawn into EDTA vacutainer tubes for subsequent immunostaining, which was initiated within the hour of each blood draw. A sample of the blood was used for plasma banking at -80°C for subsequent testing of cytokine profile.

Below is a simplified diagram illustrating the involvement of each participant, where all study participants were tested on two different clinic days separated by a 1-week wash-out period.

### Consumable test product

The active test product, UP360® is a blend of extracts from *Aloe vera*, the medicinal mushroom *Poria cocos*, and an aqueous extract from *Rosmarinus officinalis* (rosemary) (**Table 1**) and is described in this paper as the APR extract. It contained a formulation of *Aloe vera* gel powder, *Poria cocos* and rosemary extracts. Briefly, fresh *Aloe* leaves were washed, and the outer skin was removed. The inner leaf gel was enzymatically digested, filtered through activated charcoal, and concentrated by low pressure evaporation followed by dehydration. The lyophilizate contained no less than 10% polysaccharides with a molecular weight distribution between 50–200 kDalton (kDa) (average molecular weight at 80 kDa). An ethanol/water extract of rosemary leaves contained no less than 30% rosmarinic acid. *Poria cocos* scleroticum was ground to a powder and extracted with ethanol and water resulting in a precipitate with no less than 20% polysaccharides. The formulation of UP360 contained *Aloe* leaf gel powder, *Poria* extract, and rosemary leaf extract at a ratio of 3:6:1 by weight. The placebo contained microcrystalline cellulose and magnesium stearate. Both products were manufactured and encapsulated in opaque capsules by Acenzia 124 (Tecumseh, Ontario, Canada).

The active product and placebo were provided by Unigen Inc., Tacoma WA USA, encapsulated in veggie capsules with 500 mg/capsule. The appearance of the active product and placebo were identical. During the study, each participant consumed a single dose of placebo and a single dose of the APR extract on different clinic visits, in the presence of clinic staff. Therefore, the compliance with respect to product consumption was 100%.

#### Reagents

Blood collection tubes containing EDTA (pink-top, 6mL vacutainer tubes) and butterfly needles were purchased from Becton-Dickinson (Franklin Lakes, NJ, USA). Attune Focus fluid, Performance tracking beads, wash and shut-down solutions, de-bubble buffer, and the 2 lysing buffers, High Yield Lysing buffer™ and Cal-Lyse™ whole-blood lysing solution, were purchased from Thermo Fisher Scientific (Waltham, MA, USA). Monoclonal antibodies described below were obtained from Thermo Fisher Scientific (Waltham, MA, USA), except the CD25-Brilliant Violet421 antibody that was obtained from BD Biosciences (Franklin Lakes, NJ, USA). Customized Bio-Plex Pro™ human cytokine arrays were purchased from Bio-Rad Laboratories Inc. (Hercules, CA, USA).

#### Immune cell evaluation by flow cytometry

From each blood sample, whole blood was used to evaluate changes to immune cell numbers and activation status. All immunostaining was performed in triplicate on fresh blood within an hour of drawing the blood. Three different panels of monoclonal antibodies were used: 1) NK panel: A panel focused on NK cells, NKT cells, T cells, and monocytes and activation markers on those cell types, 2) T cell panel: A panel focused on CD8+ versus CD4+ T lymphocytes and also evaluating CD45 isoforms on each subset of T cells as well as B lymphocytes, and 3) A gamma delta (γδ) T cell panel, focused on γδT lymphocytes and activation markers on those cells.

The NK cell panel made use of the following monoclonal antibodies: CD3-SuperBright702 (clone OKT3), CD25-SuperBright600 (clone BC96), CD56-Phycoerythrin (clone CMSSB), CD57-eFluor450 (clone TB01), and CD69-Fluorescein isothiocyanate (clone FN50).

The T cell panel made use of these monoclonal antibodies: CD4-SuperBright702 (clone SK3), CD8-Fluorescein Isothiocyanate (clone OKT8), CD19-SuperBright600 (clone SJ25C1), DC45RA-SuperBright436 (clone HI100), and CD45R0-Phycoerythrin (clone UCHL1).

The γδT cell panel made use of these monoclonal antibodies: γδT cell antibody-Fluorescein isothiocyanate (clone SK7), CD5-SuperBright702 (clone UCHT2), CD25-Brilliant Violet421 (clone 2A3), CD56-SuperBright600 (clone TULY56), and CD69-Phycoerythrin (clone FN50).

For the NK and γδT cell panels, triplicate samples of 100 μL heparinized whole blood were stained with the mix of monoclonal antibodies listed above, incubated for at least 15 minutes at room temperature in the dark, followed by addition of 2 mL of High-Yield Lysing buffer, mixing, and incubation for at least 10 minutes at room temperature in the dark. Samples were transferred to 2-mL deep-well 96-well plates and samples were acquired by flow cytometry within 4 hours of staining.

For the T cell panel, triplicate samples of 100 μL heparinized whole blood were stained with the mix of monoclonal antibodies listed above, incubated for at least 15 minutes at room temperature in the dark, after which 100 μL Cal-Lyse buffer was added and the samples were mixed carefully. After another 10 minutes of incubation the red blood cells were lysed by addition of 1 mL of distilled water and mixed by vortexing. Samples were stored in the dark and acquired by flow cytometry within 24 hours.

All samples were acquired using an acoustic-focusing Attune™ Nxt flow cytometer (Thermo Fisher Scientific), using a microplate-based Autosampler. Data analysis utilized gating on forward/side scatter to identify lymphocyte and monocyte populations, followed by gating based on NK and T cell markers. Each cell subset was then analyzed for expression levels of the activation markers.

#### Levels of cytokines, chemokines, and growth factors

Serum samples from all blood draws were used for evaluation of changes to blood levels of 27 cytokines and chemokines, quantified using Bio-Plex protein arrays (Bio-Rad Laboratories Inc.) and utilizing xMAP technology (Luminex, Austin, TX, USA). The following markers were tested: IL-1β, IL-1ra, IL-2, IL-4, IL-5, IL-6, IL-7, IL-8, IL-9, IL-10, IL-12 (p70), IL-13, IL-15, IL-17, Eotaxin, Basic FGF, G-CSF, GM-CSF, IFN-γ, IP-10, MCP-1 (MCAF), MIP-1α, MIP-1β, PDGF-BB, RANTES, TNF-α, and VEGF.

#### Statistical analysis

CONSORT guidelines were followed to report the results from this clinical trial. Average and standard deviation for each data set was calculated using Microsoft Excel (Microsoft Corporation, Redmond, WA, USA). Post-consumption changes from baseline to later assessments were evaluated by between-treatment analysis for each time point. This allowed evaluation of changes on the day a person consumed the active product, in context of the person’s circadian changes on the day he/she consumed placebo. The evaluation used within-subject analysis and the two-tailed paired t-test where statistical significance was set at P<0.05, and a high level of significance P<0.01.

## Results

### Study population and compliance

The demographic characteristics for the study participants are shown in **Table 2**. All 11 study participants completed the study participation with full compliance, including adhering to similar routines and food for 12 hours prior to arrival on both clinic days, remaining calm and unstressed during the 4-hour clinic visits, consuming test products with water as instructed, and allowing the four blood draws at each visit.

**Table 2:** Demographics of the study population.

**All Participants**	**11**
Age average (years)^a^	41.3 ± 20.7
Age range (years)	19.2–73.5
BMI average (kg/m^2^)^a^	24.9 ± 4.5
BMI range (kg/m^2^)	19.8–31.5
**Females**	**5**
Age average (years)^a^	45.2 ± 22.2
Age range (years)	20.9–68.6
BMI average (kg/m^2^)^a^	24.2 ± 4.0
BMI range (kg/m^2^)	20.3–29.6
**Males**	6
Age average (years)^a^	38 ± 20.9
Age range (years)	19.2–73.5
BMI average (kg/m^2^)^a^	25.5 ± 5.1
BMI range (kg/m^2^)	19.8–31.5

^a^The average ± standard deviation is shown.

#### Cytokine profile

Changes in cytokine levels in the blood samples after consumption of the active test product UP360 were compared to changes after consuming placebo, to account for circadian changes unique to each study participant during the morning hours when the study was conducted. Eight detectable cytokines, chemokines, and growth factors are listed in **Table 3.**

**Table 3:** Cytokines, chemokines, and growth factors with detectable changes in this study.

Immune signaling molecule	Abbreviation	Produced by	Function
**Interferon gamma-induced protein-10**	IP-10 (CXCL-10)	Monocytes [69], endothelial [70]	Chemoattraction of innate immune cells
**Monocyte chemoattractant protein-1**	MCP-1 (CCL-1)	Macrophage [71], endothelial [72]	Chemoattraction of monocytes and dendritic cells
**Eotaxin**	EOX (CCL-11)	Monocytes [73], epithelium [74]	Chemoattraction of eosinophils
**Regulated upon Activation, Normal T Cell Expressed and Presumably Secreted.**	RANTES (CCL-5)	T-cells [75], epithelial cells [76]	Chemoattraction of T cells, NK cells, monocytes
**Interleukin-4**	IL-4	T cells [77], eosinophils [78]	Proliferation of activated T and B cells
**Interleukin-17**	IL-17	NKT cells [79], TH17 cells [80]	Regulates NFkB and MAP kinases
**Tumor necrosis factor-alpha**	TNF-α	Macrophages [81], NK cells [82]	Immune cell activation
**Granulocyte-Colony Stimulating Factor**	G-CSF	Stromal cells [83]	Stem cell mobilization

**Fig 3:**
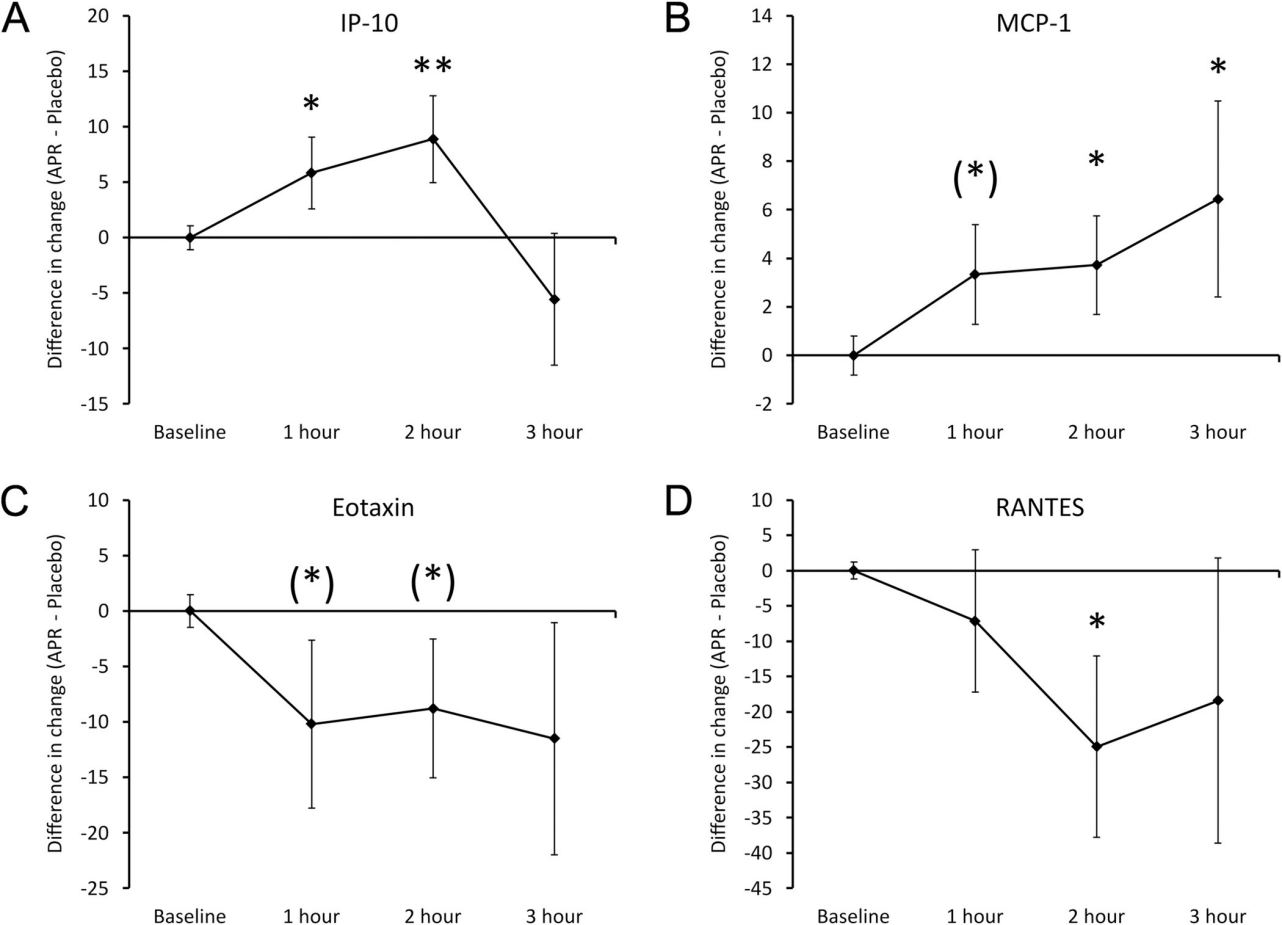
Differences in changes to serum chemokine levels within 3 hours after consumption of APR extract versus placebo. The results are shown as the group averages ± standard error of the mean of the individual percent changes from baseline after consuming APR extract where changes after consuming placebo are subtracted. A. Interferon-gamma inducing Protein-10 (IP-10): The relative increase associated with APR consumption reached statistical significance after 1 hour, and a high level of statistical significance after 2 hours. The relative increase in IP-10 levels returned to similar levels as after consuming placebo after 3 hours. B. Monocyte Chemoattractant protein-1 (MCP-1): A relative increase after consumption reached a statistical trend at 1 hour after consuming APR extract, and statistical significance after 2 and 3 hours. C. Eotaxin: A relative decrease reached statistical trends at 1 and 2 hours, but lost significance at 3 hours. D. RANTES: A 25% decrease at 2 hours reached statistical significance. Levels of statistical significance are shown on the graphs where changes from baseline to a later time point is indicated by asterisks, where p<0.10: (*), p<0.05: * and p<0.01: **.

The differences in cytokine levels after consuming APR extract versus placebo included an increase in Interferon gamma-induced protein-10 (IP-10) and Monocyte Chemoattractant protein-1 (MCP-1), reaching statistical significance after 1 hour for IP-10 and a statistical trend after 1 hour for MCP-1 (**Fig 3**). The relative changes to IP-10 levels (**Fig 3A**) reached a high level of statistical significance at 2 hours (P<0.01), after which it returned to similar levels as after consuming placebo at 3 hours. The relative changes to MCP-1 (**Fig 3B**) continued to increase up to the 2-hour and 3-hour blood draw, where the increase was statistically significant (P<0.05).

At the same time, Eotaxin and RANTES showed relative decreases (**Fig 3**). The Eotaxin levels (**Fig 3C**) reached a 10% decrease and showed a statistical trend at 1 and 2 hours (P<0.1). The relative decrease in RANTRS levels (**Fig 3D**) reached statistical significance at 2 hours (P<0.05), after which it returned to similar levels as after consuming placebo.

**Fig 4:**
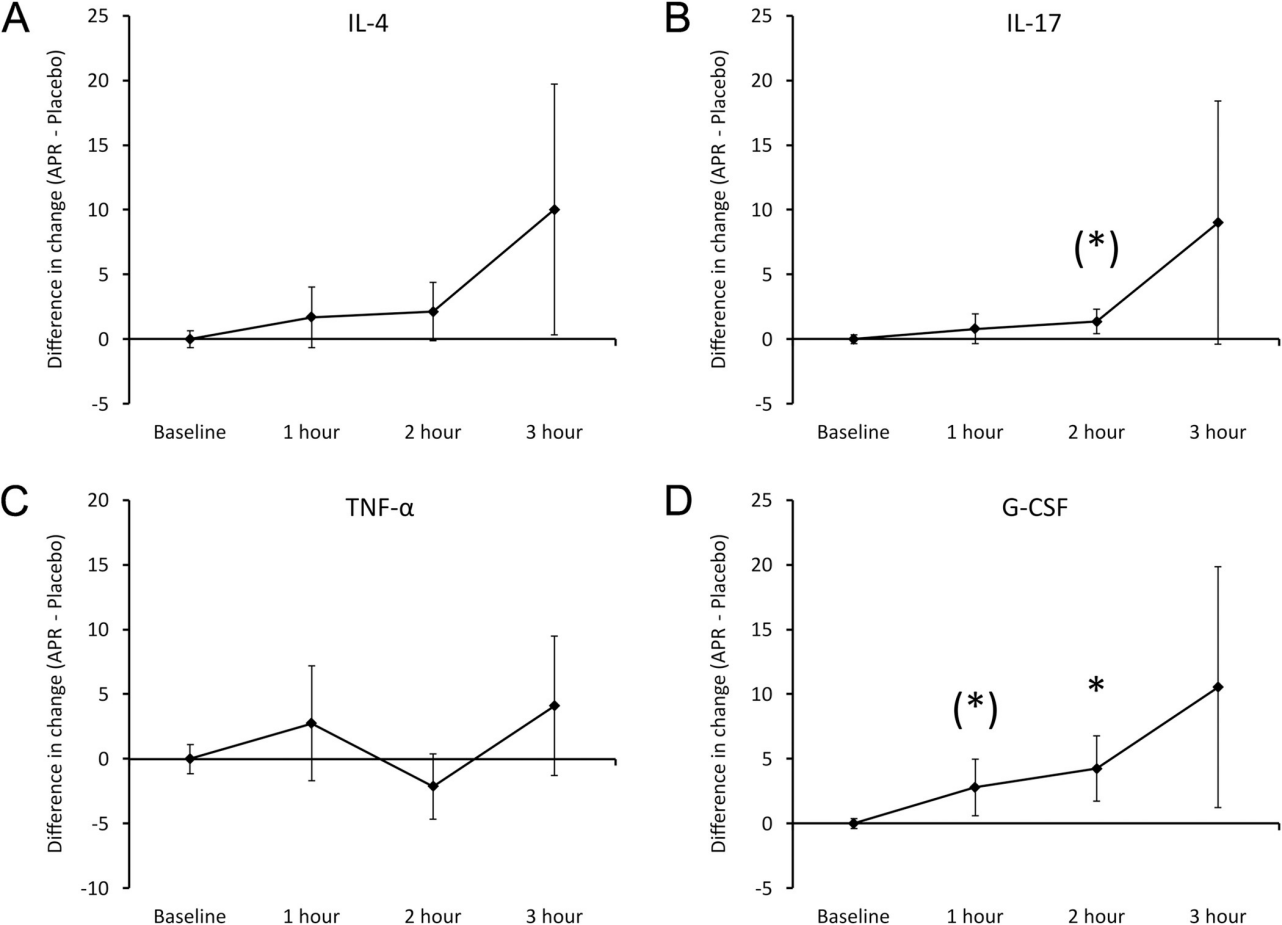
Differences in changes to serum cytokine levels within 3 hours after consumption of APR extract versus placebo. The results are shown as the group averages ± standard error of the mean of the individual percent changes from baseline after consuming APR extract where changes after consuming placebo are subtracted. A. Interleukin-4 (IL-4): A relative increase associated with APR consumption was strongest at 3 hours after consumption but did not reach statistical significance. B. Interleukin-17 (IL-17): A relative increase associated with APR consumption was strongest at 3 hours after consumption and a statistical trend was reached at 2 hours. C. Tumor Necrosis Factor-alpha (TNF-α): No changes were observed. D. G-CSF: A mild increase reached a statistical trend after 1 hour and reached statistical significance after 2 hours. Levels of statistical significance are shown on the graphs where changes from baseline to a later time point is indicated by asterisks, where p<0.10: (*), p<0.05: * and p<0.01: **.

Several other cytokines showed changes (**Fig 4**), including increased levels of IL-4 (**Fig 4A**) at 3 hours (not significant). IL-17 levels (**Fig 4B**) increased continuously, reaching a statistical trend at 2 hours (P<0.1). It was noteworthy that Tumor Necrosis Factor-alpha (TNF-a) (**Fig 4C**) did not show changes, showing that the consumption of the APR extract triggered a highly selective change to cytokine profiles, not encompassing all pro-inflammatory cytokines. The stem cell mobilizing growth factor G-CSF (**Fig 4D**) showed a relative increase that reached a statistical trend at 1 hour (P<0.1) and statistical significance at 2 hours (P<0.05).

**Fig 5:**
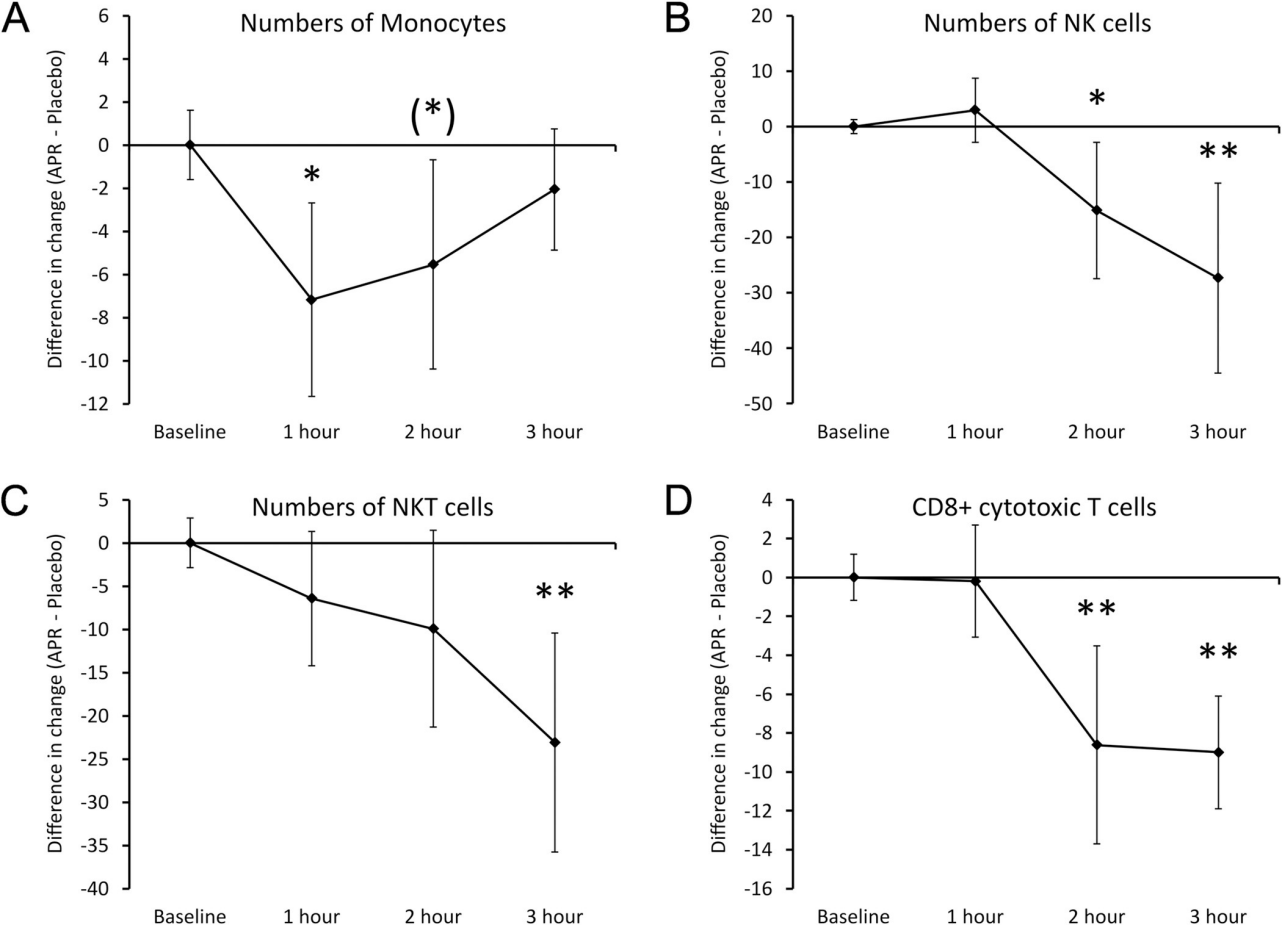
Differences in changes to immune cell trafficking reflected by the numbers of immune cells in the blood circulation within 3 hours after consumption of APR extract versus placebo. The results are shown as the group averages ± standard error of the mean of the individual percent changes from baseline after consuming APR extract where changes after consuming placebo are subtracted. A. Monocyte numbers: A mild and transient reduction was seen at 1 hour and returned to similar levels as after consuming placebo at 3 hours. The reduction at 1 hour was statistically significant. B. NK cell numbers: A robust change was seen after 2–3 hours, where the relative reduction in NK cells in the blood circulation associated with APR consumption was strongest at 3 hours after consumption. C. NKT cell numbers: A relative reduction after consuming the APR extract compared to placebo reached a high level of statistical significance at 3 hours. D. Numbers of CD8+ cytotoxic T cells: A mild reduction in the blood circulation reached high levels of significance at 2 and 3 hours. Levels of statistical significance are shown on the graphs where changes from baseline to a later time point is indicated by asterisks, where p<0.10: (*), p<0.05: * and p<0.01: **.

**Fig 6:**
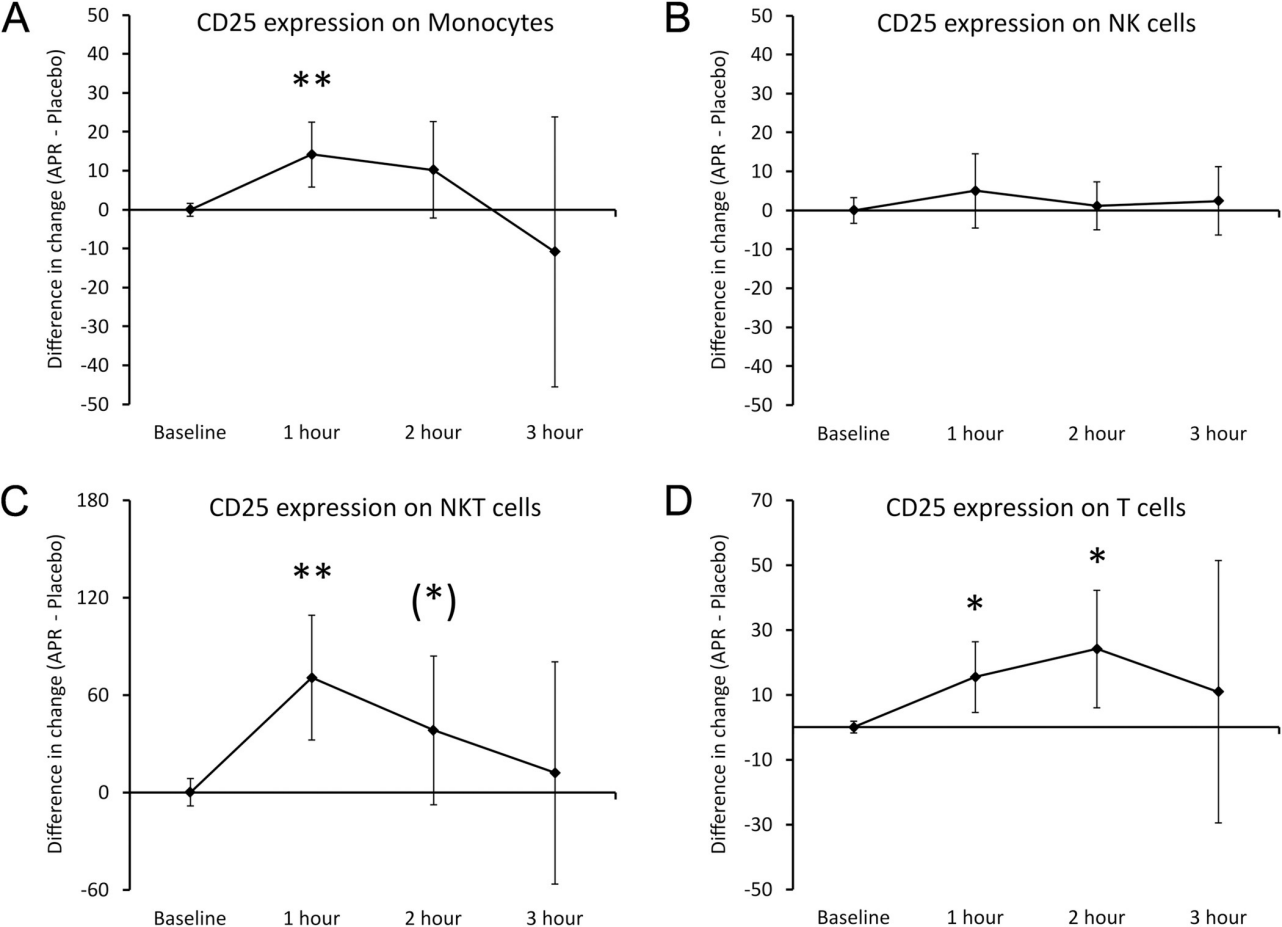
Differences in changes to expression of the CD25 activation marker on immune cells within 3 hours after consumption of APR extract versus placebo. The results are shown as the group averages ± standard error of the mean of the individual percent changes from baseline after consuming APR extract where changes after consuming placebo are subtracted. A. CD25 expression on monocytes: A relative increase reached a high level of statistical significance at 1 hour and returned to similar levels as after consuming placebo at 3 hours. B. CD25 levels on NK cells: There were no differences after consuming the APR extract compared to placebo. C. CD25 expression on NKT cells: A large increase reached a high level of statistical significance at 1 hour, remained a statistical trend at 2 hours and returned to similar levels as placebo at 3 hours. D. CD25 expression on T cells: An increase was statistically significant at 1 and 2 hours, returning to similar levels as after consuming placebo at 3 hours. Levels of statistical significance are shown on the graphs where changes from baseline to a later time point is indicated by asterisks, where p<0.10: (*), p<0.05: * and p<0.01: **.

Immune surveillance. Consumption of APR extract triggered rapid changes to immune surveillance, as evidenced by rapid changes to the numbers of specific types of immune cells in the blood circulation (**Fig 5**). At 1 hour after consumption of a single dose of 500 mg, the levels of monocytes (**Fig 5A**) were significantly lower than after consuming placebo (P<0.05), suggesting an increased trafficking of monocytes out of the blood circulation into tissue. After 3 hours, the levels of monocytes in the blood circulation had returned to similar levels as after consuming placebo. Several other cell types also showed changes, but at a different timeline. The relative numbers of CD3- CD56+ NK cells and of CD3+ CD56+ NKT cells showed a decline after 2 hours, where the reduced levels of NK cells (**Fig 5B**) reached statistical significance at 2 hours (P<0.05).The reduced levels of NK cells (**Fig 5B**) and NKT cells (**Fig 5C**) reached a high level of statistical significance at 3 hours (P<0.01). In addition, the numbers of CD8+ cytotoxic T cells (**Fig 6D**) were also reduced, reaching high levels of significance at 2 and 3 hours (P<0.01).

The expression of the 2 activation markers CD25 and CD69 were evaluated on monocytes, NK cells, NKT cells, and T cells (**Fig 6**). A highly significant increase (P<0.01) in CD25 was detected on monocytes (**Fig 6A**) at the 1-hour time where the monocyte numbers were reduced, showing that among the monocytes remaining in the blood circulation some were activated. In contrast, there was no change in CD25 expression on NK cells (**Fig 6B**). NKT cells also showed a rapid increase in CD25 expression (**Fig 6C**), reaching a high level of statistical significance at 1 hour (P<0.01) and remaining a statistical trend at 2 hours (P<0.1), after which it returned to similar levels as after consuming placebo. T cells showed a more gradual increase in CD25 expression (**Fig 6D**), reaching statistical significance at 1 and 2 hours (P<0.05), after which it returned to similar levels as after consuming placebo.

**Fig 7:**
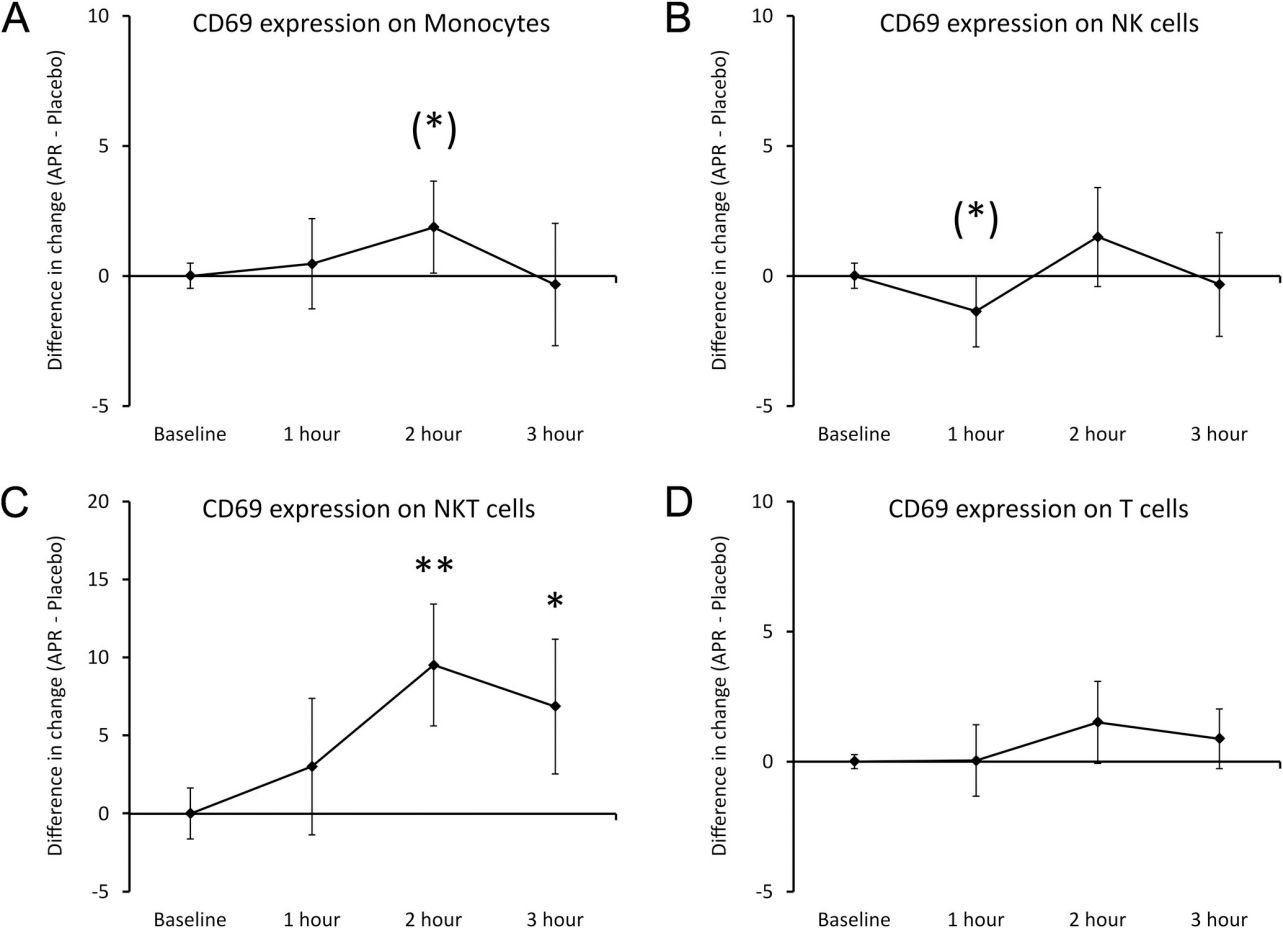
Differences in changes to expression of the CD69 activation marker on immune cells within 3 hours after consumption of APR extract versus placebo. The results are shown as the group averages ± standard error of the mean of the individual percent changes from baseline after consuming APR extract where changes after consuming placebo are subtracted. A. CD69 expression on monocytes: A relative increase was seen at 2 hours and reached a statistical trend. B. CD69 expression on NK cells: A relative decrease in was seen at 1 hour and reached a statistical trend. C. CD69 expression on NKT cells: A large increase reached a high level of statistical significance at 2 hours and remained statistically significant at 3 hours. D. CD69 levels on T cells: There were no differences after consuming the APR extract compared to placebo. Levels of statistical significance are shown on the graphs where changes from baseline to a later time point is indicated by asterisks, where p<0.10: (*), p<0.05: * and p<0.01: **.

The CD69 expression showed mild increase on monocytes (**Fig 7A**), reaching a statistical trend at 2 hours (P<0.1) and at 3 hours returned to similar levels as after consuming placebo. NK cells showed mild change in CD69 expression (**Fig 7B**), with a decrease reaching a statistical trend at 1 hour (P<0.1). The strongest changes to CD69 expression were observed on NKT cells (**Fig 7C**), reaching a high level of statistical significance at 2 hours (P<0.01) and maintaining statistical significance at 3 hours (P<0.05). There was no significant change in CD69 expression on T cells (**Fig 7D**).

#### Gamma delta T cells

**Fig 8:**
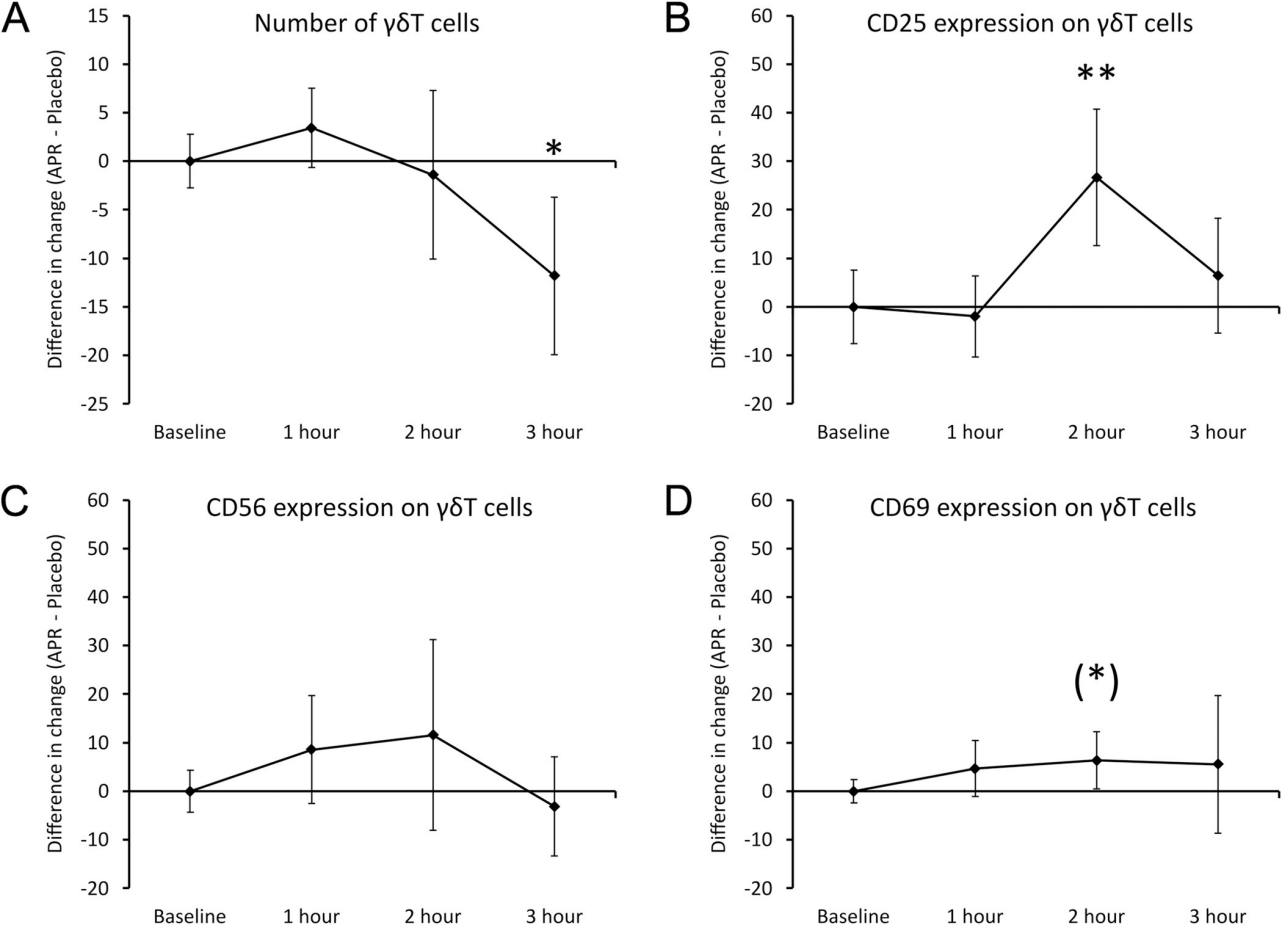
Differences in changes to γδT cells within 3 hours after consumption of APR extract versus placebo. The results are shown as the group averages ± standard error of the mean of the individual percent changes from baseline after consuming APR extract where changes after consuming placebo are subtracted. A. Numbers of γδT cells: A relative decrease associated with APR consumption was strongest at 3 hours after consumption where the difference between APR and placebo reached statistical significance. B. CD25 expression on γδT cells: A relative increase associated with APR consumption was strongest at 2 hours after consumption and reached a high level of statistical significance. C. CD56 expression on γδT cells: There were no changes. D. CD69 expression on γδT cells: A mild increase at 2 hours after APR consumption reached a statistical trend. Levels of statistical significance are shown on the graphs where changes from baseline to a later time point is indicated by asterisks, where p<0.10: (*), p<0.05: * and p<0.01: **.

The number and activation status of γδT cells changed significantly after consuming APR extract compared to placebo (**Fig 8**). There was a relative decrease in the numbers of γδT cells (**Fig 8A**) in the blood circulation at 3 hours after consuming the APR extract when compared to placebo; this decrease was statistically significant (P<0.05). This change was preceded by a highly significant increase in the CD25 activation marker on the γδT cells (**Fig 8B**) at 2 hours after consumption (P<0.01). In contrast, there were no differences in CD56 expression (**Fig 8C**) when comparing results after consuming the APR extract to placebo. There was a mild increase reaching a statistical trend (P<0.1) in the CD69 activation marker on the γδT cells (**Fig 8D**) in the blood circulation at 2 hours after consuming the APR extract when compared to placebo.

#### Antioxidant enzymes

**Fig 9:**
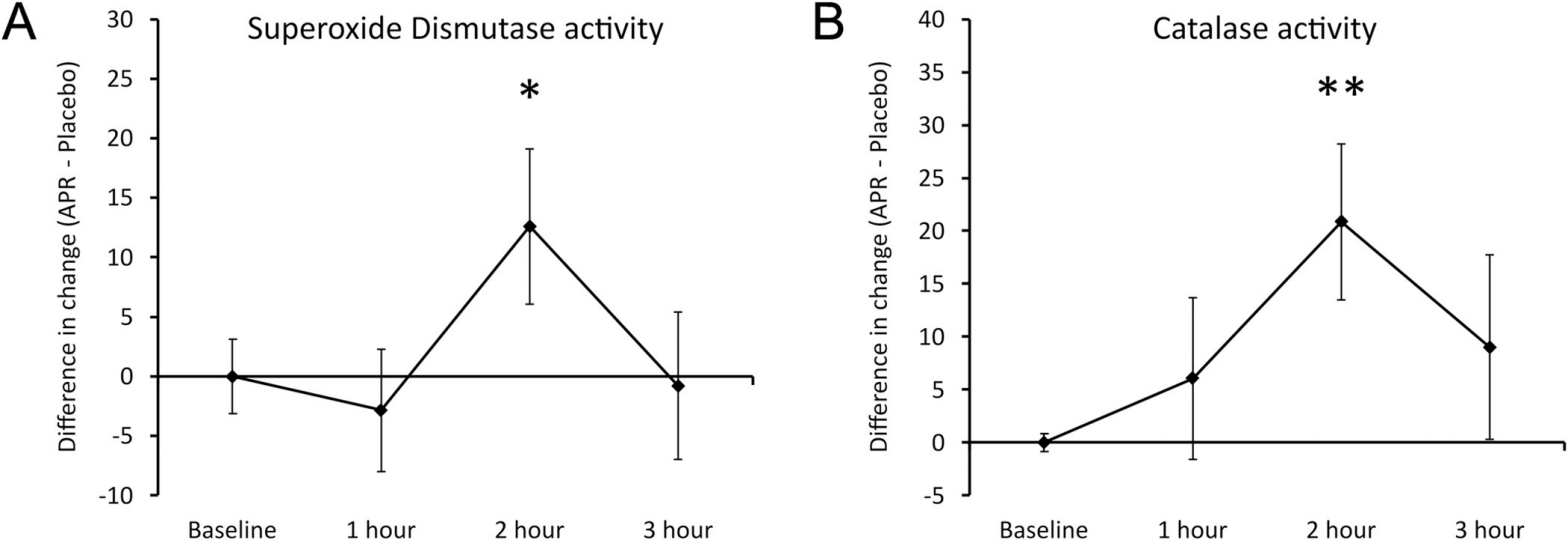
Differences in changes to antioxidant enzymes within 3 hours after consumption of APR extract versus placebo. The results are shown as the group averages ± Standard Error of the Mean of the individual percent changes from baseline after consuming APR extract where changes after consuming placebo are subtracted. A. The relative increase in the enzymatic activity of Superoxide Dismutase associated with APR consumption was strongest at 2 hours after consumption where the difference between APR and placebo reached statistical significance. B. The relative increase in the enzymatic activity of catalase associated with APR consumption was also strongest at 2 hours after consumption and reached a high level of statistical significance. Levels of statistical significance are shown on the graphs where changes from baseline to a later time point is indicated by asterisks, where p<0.10: (*), p<0.05: * and p<0.01: **.

Consumption of APR extract was associated with increased activity of the cellular antioxidant protection via 2 specific enzymes: Superoxide Dismutase (SOD) and catalase (**Fig 9**). The consumption of APR extract triggered significant increase in SOD at 2 hours (P<0.05) (**Fig 9A**) and a highly significant increase in catalase activity at 2 hours (P<0.01) (**Fig 9B**).

## Discussion

Nutraceutical effects on immune surveillance are mediated by multimodal effects after consumption, including absorption of bioavailable compounds, and direct immune modulation by compounds remaining in the gut lumen through activation of dendritic cells in the mucosal barrier [35]. Rapid neuroendocrine communication signals allow systemic effects to follow in a sequential manner.

The effects of consuming the blend of *Aloe*, *Poria* and rosemary (APR extract) were studied using a placebo-controlled cross-over design to capture changes in immune status in context to the normal circadian changes seen when the active product was not consumed. The changes after consuming the APR extract included rapid chemokine and cellular signals, followed by more sustained effects. The earliest events documented at 1 hour after consumption were a significant increase in Interferon gamma-induced protein 10 (IP-10) and monocyte chemoattractant protein-1 (MCP-1), possibly produced by gut mucosal macrophages after activation by compounds in the APR extract. This showed similar magnitude for both genders, and was associated with a mild but significant reduction in monocyte numbers in the blood circulation, suggesting increased immune surveillance into tissue by a small percentage of the blood monocytes. Both effects were rapid and transient, since at 3 hours after consumption, the levels showed no difference from the levels after consuming placebo.

In contrast, at the same time, the two inflammatory cytokines Eotaxin and RANTES were reduced in the blood circulation; the underlying regulating mechanisms are unknown but may involve reduced production of these cytokines by tissue-resident fibroblasts and T cells. This suggests a reduction in inflammatory interference, allowing immune cells in the blood circulation to better sense the chemotactic signals from tissue, triggering increased immune cell migration out of the blood circulation. It is important to note that the cytokine effects triggered by consumption of the APR extract were highly selective, showing strong and highly significant changes for some cytokines, and no change for other inflammatory cytokines such as TNF-α.

Increased immune cell surveillance occurred in sequential waves, with the monocytes migrating at 1 hour after consumption and several sub-populations of killer cells showing migration at 2–3 hours. Monocytes remaining in the blood circulation included cells with a significantly increased level of CD25 on the cell surface, possibly due to monocytes that had attempted to extravasate, and became activated in the process of engaging with adhesion molecules on the endothelium [3, 4].

**Fig 10:**
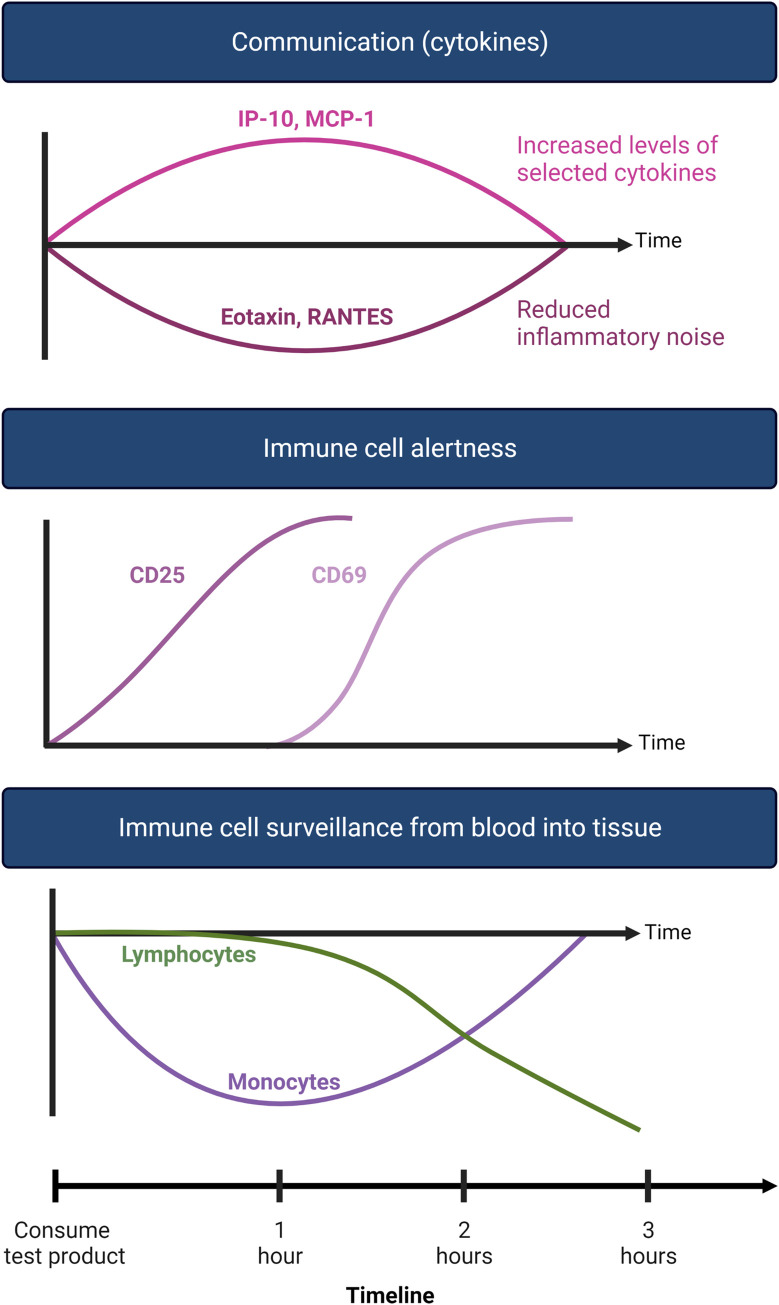
Diagram showing the events associated with consuming a single dose of APR extract when compared to consuming a single dose of placebo.

Numbers of natural killer T (NKT) cells were significantly reduced at 3 hours after consuming the APR extract, indicating an increase in immune surveillance (**Fig 10**). The NKT cells showed increased expression of the CD25 activation marker at 1 hour, reaching a high level of statistical significance, suggesting increased immune alertness. The expression of the CD69 activation marker was increased significantly at 2 hours after consumption, suggesting NKT cells were on a higher alert and primed for performing a cytotoxic attack on target cells. Similar effects were seen for T cells, including CD8+ cytotoxic T cells and γδT cells. This indicates that cellular activation was an early event, preceding migration of NKT cells. The increased activation and migration of γδT cells may suggest a transient increase in γδT cell surveillance of the gut mucosa.

**Fig 11:**
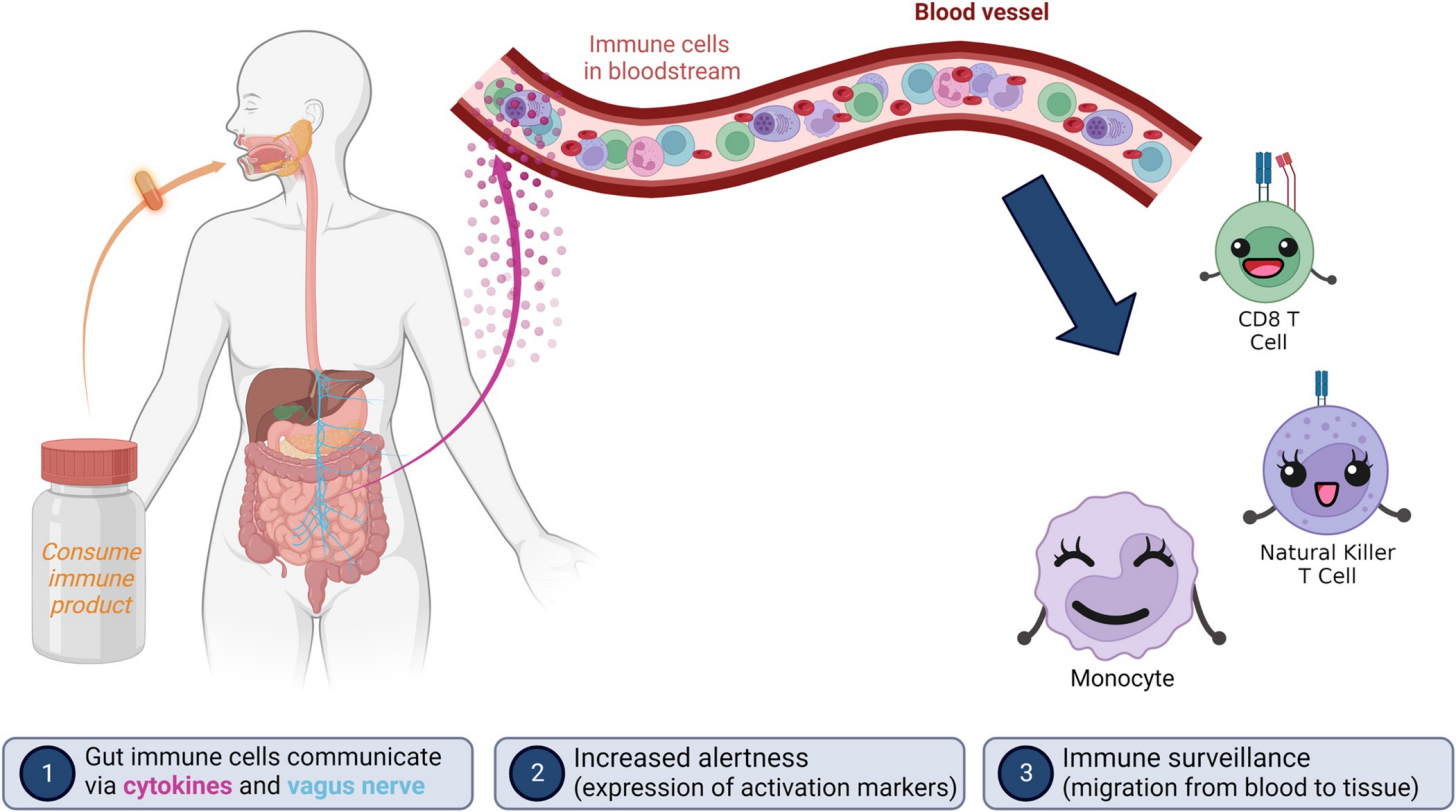
Diagram showing the 3 different waves of immune modulating events after consuming an immune-modulating product.

The effects of consuming the APR extract on immune surveillance were highly selective, and were limited to innate effector cell types, including monocytes, NKT cells, CD8+ T cells, and γδT cells. NK cell numbers decreased, but there was not a change in CD25 expression on NK cells, and only a mild decrease reaching statistical trend in CD69 expression on NK cells was observed (**Fig 11**). Other cell types were not affected in this acute study, including B lymphocytes, suggesting that the immediate mechanisms were selective to monocytes and specific types of killer cells. This is in line with the effects on NK cells we observed at 1 hour after consuming a low molecular weight extract from bovine colostrum [61], and the yeast fermentate EpiCor [38]. This points to opportunities to combine nutraceuticals with different effects or select specific nutraceuticals for specific types of immunological challenges.

The placebo-controlled cross-over study design, combined with the fixed time of the day in which the study took place, allowed us to evaluate the normal circadian changes for each study participant on the morning when they consumed placebo, and compare to changes on the day they consumed the APR extract.

This study was conducted in healthy adults in the absence of an immune challenge and observed the increases in migration and alertness of the affected types of innate immune cells. We speculate that in the presence of a pathogenic insult the increased activation status and migration of innate immune cells would be accompanied by increased cytotoxic activity, leading to increased inflammatory and free radical stress. In that context, it is important to note that the consumption of the APR extract also was associated with anti-inflammatory properties, specifically a significant increase in the activity of two antioxidant enzymes, superoxide dismutase and catalase. In a situation involving an immune challenge this increased antioxidant protection would limit the tissue damage associated with the active immune response and support an accelerated return to homeostasis. There was also a statistically significant increase at 2 hours in the growth factor G-CSF, a growth factor associated with stem cell mobilization [84], suggestive of an increase in other synergistic restorative functions after consuming the APR extract.

This clinical study is the first to document rapid effects of APR extract on rapid changes to immune surveillance and antioxidant protection in humans. Consuming the APR extract showed highly selective support of the innate immune system. At the cellular level, the initial events included increased immune cell alertness through expression of the activation marker CD25, followed at 2 hours by the expression of CD69 which demonstrates an increased preparedness for performing cytotoxic immune function [85, 86]. This was then followed by increased immune surveillance at 3 hours for several types of cytotoxic innate immune cell types.

Previous research on the APR extract showed rapid effects in a mouse model, involving 100% survival after 36 hours after acute lung injury, in contrast to lower survival rates in mice treated with any of the single ingredients [87]. A second study in mice showed increased antioxidant capacity through increased superoxide dismutase levels [S1 File]. Long-term consumption of the APR extract in mice was associated with increased levels of circulating cytotoxic NK and T cells, including γδT cells mice [87]. It is interesting that consumption of the APR extract leads to rapid increase in immune surveillance of CD8+ cytotoxic T cell and g γδT cells, and with long-term consumption the result is an increase in the levels of these cells in the blood circulation. The combined findings suggest increased protection of mucosal, lung, and skin barriers, both through increased levels and increased surveillance by multiple types of innate immune cells, including γδT cells. Furthermore, the immediate anti-inflammatory effects were seen by increased activity of superoxide dismutase and catalase, whereas long-term consumption showed additional effects including a significant reduction in TNF-α after 7 days [S1 Data]. Long-term consumption in humans in context of an immune challenge in the form of involving influenza vaccination showed increased levels of influenza-specific immunoglobulin production [88].

Although the immune evaluations performed in this clinical trial were comprehensive and well-controlled, a future study should also include ex vivo challenges, where immune cells from the blood samples can be exposed to bacterial and viral mimetics in vitro, and the responses tested via cytokine profiling. In addition, based on the increased levels of the stem cell-mobilizing growth factor G-CSF, it would be of interest to document changes to stem cell mobilization and surveillance after consuming the APR extract. This would follow the same study design as previously used to document acute and transient stem cell effects by other nutraceuticals [62–64].

The opportunity for supporting natural immune surveillance activities by nutraceutical methods has many implications, including preventive health and pro-active immune support. The benefits of consuming immune-supportive nutraceuticals could extend to people experiencing acute and chronic health problems. It is also an opportunity to mitigate or reduce the negative impact of psychological, physical, and environmental stress on immune health, as we see concerted biological communication and increased immune alertness and surveillance after consuming the APR extract. This should be the focus of additional research, such as reduction of disease severity in people with acute and chronic viral infections, to document whether nutraceutical products, such as the APR extract, could be effective tools to support and accelerate immune defense activity as well as repair and reduction of inflammation.

## Supporting information

S1 Checklist CONSORT 2010 checklist of information to include when reporting a randomised trial*. (PDF)

S1 File An aloe-based composition constituted polysaccharides and polyphenols protected mice against d-galactose induced immunosenescence. (DOCX)

S1 Data (XLSX)

S2 Data NIS labs protocol 181–002. Rapid immune modulating effects: Clinical proof-of-concept study.
(PDF)
