# Supplementary material for: Rapid increase in immune surveillance and expression of NKT and γδT cell activation markers after consuming a nutraceutical supplement containing Aloe vera gel, extracts of Poria cocos and rosemary. A randomized placebo-controlled cross-over trial
Source: PLoS One. 2023 Sep 12;18(9):e0291254. doi: 10.1371/journal.pone.0291254 (PMC10497150; doi:10.1371/journal.pone.0291254)
Supplement: S1 File — (DOCX) [file pone.0291254.s002.docx]

**Title:** An aloe-based composition constituted polysaccharides and polyphenols protected mice against d-galactose induced immunosenescence

**Abstract**

Immunosenescence and inflammaging are age-associated dysregulation of the immune system that predispose the elderly to an increased susceptibility to infection and poor vaccine response. Natural bioactives such as polysaccharide and polyphenols from botanicals are known for their immune modulation activities. Here we evaluated a standardized aloe-based composition, UP360 (constitute of polysaccharides from *Aloe barbadense* and *Poria cocos* and polyphenols from *Rosemary officinalis*) as a natural nutritional supplement for a balanced immune response in d-galactose induced accelerated aging mouse model. Immunosenescence was induced by prolonged subcutaneous injection of d-galactose at the dose of 500 mg/kg/day to CD-1 mice. The aloe-based composition was administered at oral doses of 200 mg/kg and 400 mg/kg to the mice starting the 5^th^ week of d-galactose injection. Study lasted for a total of 9 weeks. Two weeks before necropsy, all mice were given a quadrivalent influenza vaccine at 3µg/animal via intramuscular injection. A set of d-gal mice with treatment at 400 mg/kg/day was kept without vaccination. Whole blood, serum, spleen homogenate and thymus tissues were used for analysis. The Aloe-based composition, UP360, was found to show reversal of immunosenescence as evidenced by stimulation of innate and adaptive immune responses (increased IgA, increased CD3+ T cells, CD4+ Helper T cells, CD8+ Cytotoxic T cells, NKp46+ Natural Killer cells, and TCRγδ+ Gamma delta T cells), augmentation of antioxidant capacity (increased SOD and Nrf2) and preservation of key immune organs, such as thymus, from aging-associated damage of d-gal mice. These findings demonstrated the ability of the Aloe based composition to aid in activating and maintaining homeostasis of the immune system both during active infections and as a preventive measure to prime the immune system against infection. These data warrant further clinical study to explore the potential application of the aloe-based composition as an adjunct nutritional supplement for a balanced immune response.

**Keywords**: Immunosenescence, aging, polysaccharides, polyphenols, d-galactose
