# Supplementary material for: Rapid increase in immune surveillance and expression of NKT and γδT cell activation markers after consuming a nutraceutical supplement containing Aloe vera gel, extracts of Poria cocos and rosemary. A randomized placebo-controlled cross-over trial
Source: PLoS One. 2023 Sep 12;18(9):e0291254. doi: 10.1371/journal.pone.0291254 (PMC10497150; doi:10.1371/journal.pone.0291254)
Supplement: S2 Data — Rapid immune modulating effects: Clinical proof-of-concept study. (PDF) [file pone.0291254.s004.pdf]

APPROVED
AUG 1 3 2021
ARGUS IRB, INC.

NIS Labs Protocol 181-002. Rapid immune modulating effects: Clinical proof-of-concept study.

Table of Contents

1 Purpose 2
2 Study 2
2.1 Clinical Study Design 2
2.2 Outcome Measures 3
3 Study Population 3
3.1 Subjects 3
3.2 Inclusion Criteria 3
3.3 Exclusion Criteria 4
3.4 Consumable Test Products 5
3.1 Study environment 5
3.2 Study Procedures 5
3.2.1 Explanation of clinical study procedures 5
3.2.2 Table Over Study Procedures 7
3.2.3 Blood draws 8
3.3 Testing - Immune cell trafficking and status of immune alertness 8
3.3.1 NK/T cell panel 10
3.3.2 T/B cell panel 10
3.3.3 Gamma/Delta T cell panel with activation markers 11
4 References 11

NIS Labs Protocol 181-002. Rapid immune modulating effects: Clinical proof-of-concept study.

1 Purpose

The goal for this clinical proof-of-concept study is to compare acute immune effects of a novel nutraceutical blend to a placebo.

2 Study

2.1 Clinical Study Design

For this clinical study, human subjects will be tested following an **established placebo-controlled, randomized, double-blinded, cross-over study design.** Specifically, the study design has been used in previous clinical studies on immune modulating products including the yeast-based fermentate Epicor,[1] a bovine colostrum-based peptide- and oligosaccharide-rich extract Immunel,[2] the algae-based extract for stem cell support StemEnhance,[3] and an aloe-based folk medicine formulation from Madagascar.[4] Recently, the NIS Labs' team also published on changes to lymphocyte trafficking, specifically stem cell subsets, using this study design when consuming placebo versus a polyphenol-rich extract from Sea Buckthorn from Tibet.[5]

Below is a simplified diagram illustrating the involvement of each human subject, where each study participant will be tested on two different clinic days.

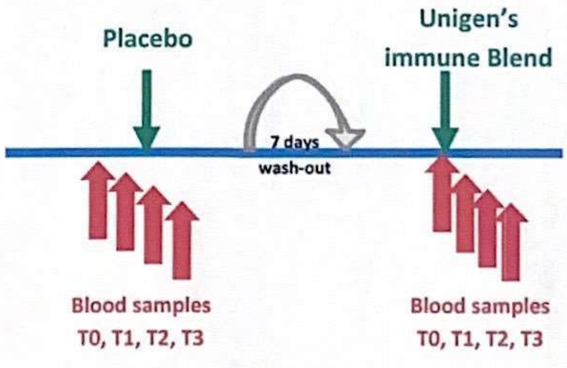

APPROVED
AUG 13 2021
ARGUS IRB, INC.

Figure 1. Diagram showing the involvement of each human subject. Study participants will be tested on two different clinic days. The sequence of test products shown here is an example only since the sequence will be randomized.

The test parameters we will evaluate do not necessarily stay constant, even over a few hours, since they are related to people's metabolism, individual circadian rhythms, and other normal physiological parameters. Therefore, studies of this nature must include a placebo test day, allowing *within-subject* analysis of changes between the test days for each person. This very much strengthens the data analysis from this type of study. In the absence of a placebo test day, we consider the data inconclusive since changes cannot be interpreted as being related to product intake.

In light of previous data on products such as Epicor[i] and Immunel,[ii] some differences were seen at 1 versus 2 hours, and it is ideal to perform testing at both time points. In addition, data will also be collected after 3 hours.

2.2 Outcome Measures

Primary outcome measure: Immune surveillance: Trafficking and activation of immune cells in vivo.

3 Study Population

3.1 Subjects

24 healthy subjects of either gender will be enrolled after IRB-approved, written informed consent. The inclusion/exclusion profile for a study of this nature is not trivial, and each potential study participant is carefully evaluated prior to enrollment. To minimize anticipatory stress and apprehension during initial clinic visits for the study, each study participant must either have participated in previous studies at our facility or must attend a visit where we go through the study procedures, prior to a clinical study day.

APPROVED
AUG 13 2021
ARGUS IRB, INC.

3.2 Inclusion Criteria

- Healthy adults;
- Age 18-75 years (inclusive);
- BMI between 18.0 and 34.9 (inclusive);
- Veins easy to see in one or both arms (to allow for the multiple blood draws);
- Willing to comply with study procedures, including:
  - Maintaining a consistent diet and lifestyle routine throughout the study;
  - Consistent habit of bland breakfasts on days of clinic visits;
  - Abstaining from exercise and nutritional supplements on morning of study visit;

- Abstaining from use of coffee, tea, and soft drinks for at least one hour prior to a clinic visit;
- Abstaining from music, candy, gum, computer/cell phone use, during clinic visits.

3.3 Exclusion Criteria

- Previous major gastrointestinal surgery (absorption of test product may be altered) (minor surgery not a problem, including previous removal of appendix and gall bladder);
- Taking anti-inflammatory medications on a daily basis;
- Currently experiencing intense stressful events/ life changes;
- Currently in intensive athletic training (such as marathon runners);
- Cancer during past 12 months;
- Chemotherapy during past 12 months;
- Currently treated with immune suppressant medication;
- Diagnosed with autoimmune disorders e.g. systemic lupus erythematosus, hemolytic anemia;
- Donation of blood during the study or within the 4 weeks prior to study start;
- Have received a cortisone shot within past 12 weeks;
- Immunization during last month;
- Currently taking antipsychotic, hypnotic, or anti-depressant prescription medication;
- Ongoing acute infections (including teeth, sinus, ear, etc);
- Participation in another clinical trial study during this trial, involving an investigational product or lifestyle change;
- An unusual sleep routine (examples: working graveyard shift, irregular routine with frequent late nights, studying, partying);
- Unwilling to maintain a constant intake of supplements over the duration of the study;
- Anxiety about having blood drawn;
- Women of childbearing potential: Pregnant, nursing, or trying to become pregnant;
- Known food allergies related to ingredients in active test product or placebo.

APPROVED
AUG 13 2021
ARGUS IRB, INC.

Prescription medication will be evaluated on a case-by-case basis.

3.4 Consumable Test Products

The active test product and the placebo will be provided by study sponsor. The active test product contains mushrooms, rosemary, and aloe, so we will ask potential study participants about food sensitivities to those ingredients.

On each clinic day, immediately after the baseline blood draw, participants will be given a single dose of either the active test product or a placebo, in the presence of the clinic staff. Participants will consume the capsules with water and a few bland soda crackers to stimulate digestive function.

3.1 Study environment

The study of acute changes to levels, activation status, and functionality of immune cells is not trivial. All test parameters undergo circadian changes, and are negatively affected by stress, adrenaline, certain medications, lack of sleep, and recent illness. The study participants will be instructed to call and reschedule a certain clinic day if they feel that any of these things are reasons to do so.

Also, the study environment is kept controlled for stressors. Cell phones are turned off at entry to clinic. Clinic phones and door chimes are off. Sensory input such as music, coffee/food smells, noises, etc., will be eliminated or kept to an absolute minimum. In order to keep volunteers in a state of mind we refer to as 'perpetually bored', but not falling asleep, they are offered a choice of light reading, crossword puzzles, or cards to play solitaire.

Upon arrival on the morning of each clinic day, participants will rest quietly for 1 hour prior to baseline blood draw. This resting period is crucial to gain representative baseline data. During this time, questionnaires will be completed to monitor previous meals, snacks, exercise, stressors, and recent sickness. Then a product will be fed to the participants. Three more blood samples will be drawn at 1, 2, and 3 hours after consumption.

APPROVED
AUG 13 2021
ARGUS IRB, INC.

3.2 Study Procedures

3.2.1 Explanation of clinical study procedures

In a clinical trial to monitor immune activating events, we expect a cascade of events, starting by activation of immune cells in the gut, systemic changes to cytokine levels, changes to immune cell trafficking (enhanced immune surveillance), followed by immune surveillance in

tissue throughout the body, and possibly re-entry of activated immune cells back into the blood circulation.

Blood samples offer a convenient window into the immune events happening after a product is consumed. We do not have convenient windows into what may happen at the initial gut activation, but we envision this is similar to events in vitro. We do not have windows into tissue and thus cannot monitor downstream events after immune cells migrate from blood into tissue to scavenge for microbial invaders and perform innate and adaptive types of immune responses. Therefore, we mimic this by taking some of the blood samples and challenging the immune cells ex vivo (outside the body) with microbial mimetics.

The testing described in **Section 3.3.1** aims to monitor rapid changes in the types and activation status of immune cells seen in the blood circulation. Increases versus decreases in numbers of immune cells in the blood is a measure of cellular trafficking in and out of the blood stream. We are looking for subtle events, where any systematic changes observed in a majority of the study participants after consuming the test product suggests immune activating events are induced.

APPROVED
AUG 13 2021
ARGUS IRB, INC.

3.2.2 Table Over Study Procedures

	Screen	Week 1					Week 2				
		Upon arrival	Base line	1 hour	2 hour	3 hour	Upon arrival	Base line	1 hour	2 hour	3 hour
Screening and enrollment											
Height (question)	X										
Weight (question)	X										
Medical/surgical history	X										
Health status interview	X										
Current medications	X										
Current supplements	X										
Recent illness	X										
Clinic days											
Pregnancy test (females)		X									
Height		X					X				
Weight (check BMI is in range)		X					X				
Informed consent		X									
Daily Intake Questionaire		X					X				
5-Question Questionnaire			X	X	X	X		X	X	X	X
Blood pressure			X	X	X	X		X	X	X	X
Blood draw (1 pink tube)			X	X	X	X		X	X	X	X
Feed test product			X					X			
Lab tests on clinic days											
Immune cell numbers and activation*			X	X	X	X		X	X	X	X
Plasma banking**			X	X	X	X		X	X	X	X
OTHER											
Adverse events***						X					X

**The immune phenotyping evaluates immune surveillance and cellular activation in vivo. Panels for NK cell activation, B and T cell activation, and numbers of gamma delta T cells are evaluated.*

***Plasma banking allows subsequent tests to be added later without repeating the clinical study.*

****Clinic staff takes note of anything observed that may be relevant to understanding the data. For example information a participant offers that was not prompted by the daily intake questions, or any other signs of stress, depression, or agitation.*

APPROVED
AUG 13 2021
ARGUS IRB, INC.

3.2.3 Blood draws

For each blood draw, we will obtain 1 EDTA vial for a total of 6 mL blood per blood draw. The EDTA blood will be kept refrigerated and staining for flow cytometry will be initiated within 2 hours of drawing the blood.

- A portion of the EDTA blood will be used for the assays described below:
  - **Immune surveillance:** Evaluation of absolute numbers of NK, NKT, T cells, non-NK non-T cells, and monocytes in the blood samples, reflecting changes in immune cell numbers, reflecting immune cell movements in and out of the blood stream (surveillance). The markers include **CD3**, **CD56**, and **CD57.**
  - **Immune cell activation status:** Evaluation of immune cell activation in vivo: Flow cytometry to evaluate effects on immune cell activation status, using the two activation markers **CD25** (the IL-2 receptor) and **CD69** (an antigen directly involved in NK cell function)**.**
  - **T and B cell subsets and CD45 isoform expression.**
  - **Gamma/delta T cells and activation status.**

- The remaining EDTA blood will be centrifuged, and plasma will be harvested and aliquoted before freezing at -80°C. The plasma will be used to test for **cytokine profile**, reflecting rapid changes to cytokine levels in each study participant.

3.3 Testing - Immune cell trafficking and status of immune alertness

The analysis allows us to detect if consumption of a test product leads to rapid changes in cell numbers in the circulation, and/or activates cells in vivo. Freshly drawn blood samples are used for the testing of changes in immune cell numbers and activation status. .The cells from each blood draw are assayed in both panels, each in triplicate. See also the figure below.

APPROVED
AUG 13 2021
ARGUS IRB, INC.

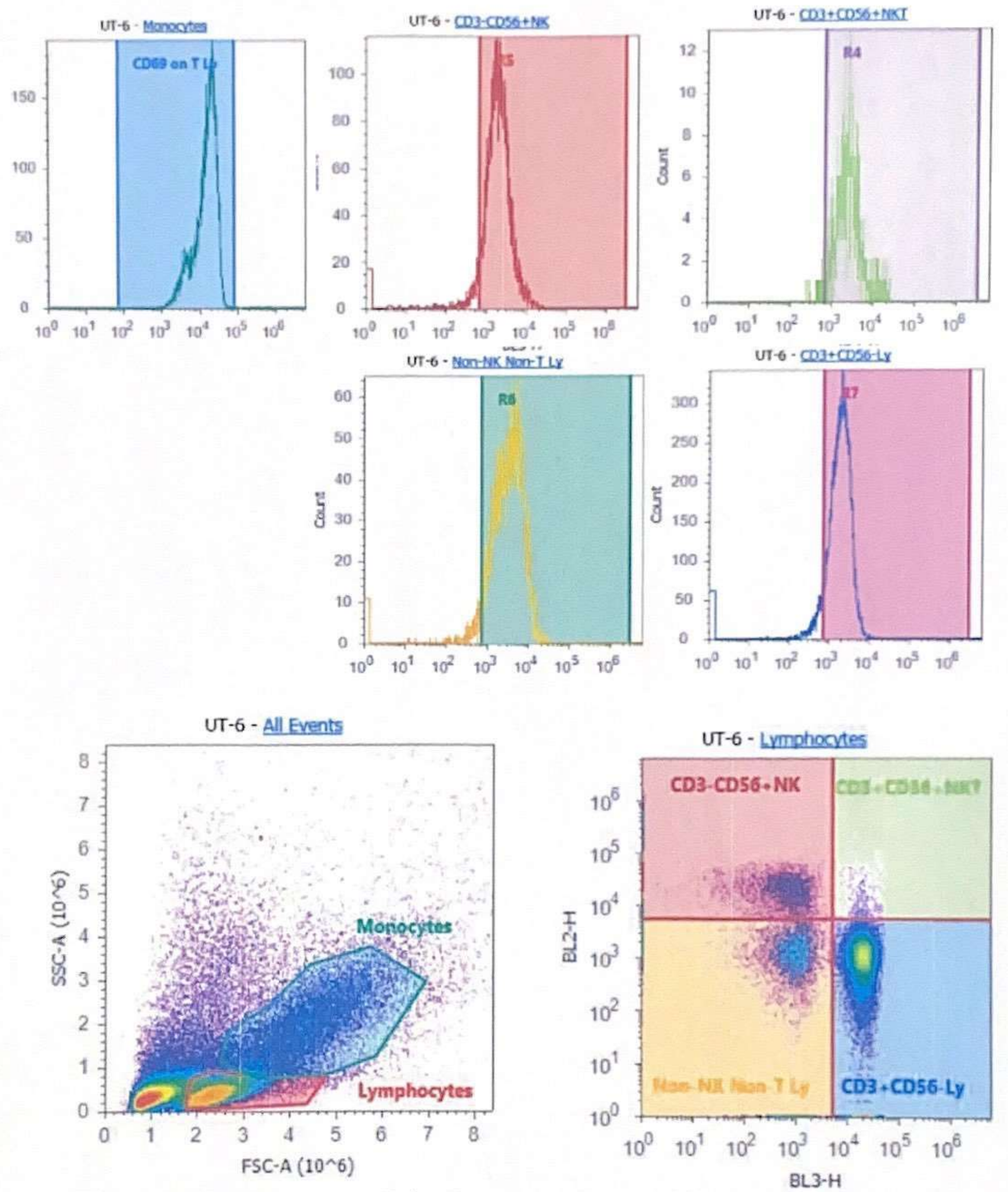

Figure 2. Flow cytometry data showing gates for lymphocytes, monocytes, and the four subsets of lymphocytes, allowing analysis of CD69 expression on all five cell types.

APPROVED
AUG 13 2021
ARGUS IRB, INC.

3.3.1 NK/T cell panel

Cells are stained with the T cell marker CD3 and the CD56 and CD57 markers, as well as the two activation markers CD69 and the interleukin-2 receptor CD25. This allows analysis of numbers of the following types of immune cells in the blood circulation at each time point in the study:

- CD3-negative, CD56-positive **NK cells**;
- CD3+ CD56+ **NKT cells**;
- CD3+ CD56- **T lymphocytes**;
- CD3-CD56- **non-NK, non-T lymphocytes**;
- CD3-CD57+ **NK cells**
- CD3- CD56+CD57+ **NK cells**
- **Monocytes** (identified by forward/side scatter profile);

During analysis, expression levels will be determined for the activation molecule **CD69** and growth factor receptor **CD25** on the surface of the cell populations listed above.

Note: Immune surveillance involves the constant recirculation of lymphocyte subsets, including NK and T cells. The trafficking shows a distinct circadian rhythm and is affected by a person's metabolic state. When comparing the acute effects of a consumable immune modulating product on immune surveillance, it is important to have a placebo control test day, to account for a given person's normal circadian changes at the time of the day of testing.

3.3.2 T/B cell panel

Cells are stained with the T cell markers CD4 and CD8, the B cell marker CD19, and co-stained with monoclonal antibodies towards CD45Ra and CD45R0 isoforms.[6] CD45Ra is expressed on naïve T cells and resting B cells. CD45R0 is expressed on memory T cells and recently activated B cells. Cells expressing both isoforms have recently been through an immune activation event.

This allows analysis of numbers of the following types of immune cells in the blood circulation at each time point in the study:

- CD4 T lymphocytes
- CD8 T lymphocytes
- CD19 B lymphocytes

APPROVED
AUG 13 2021
ARGUS IRB, INC.

Each type of lymphocyte will be analyzed for:

- CD45RA expression
- CD45Ra and CD45R0 co-expression
- CD45R0 expression

3.3.3 Gamma/Delta T cell panel with activation markers

Cells are stained with a monoclonal antibody towards the CD3/ γδ T Cell Receptor,[7 8] and co-stained with CD5. The cells are also stained for CD56 which may be expressed on some γδ T cells.[9] The 2 activation markers CD69 and CD25 will also be used. This allows analysis of numbers of the following types of immune cells in the blood circulation at each time point in the study:

Add-on panel for numbers of gamma-delta (γδ) T cells (γδTCR+ CD5-):

- CD3/ γδ T Cell Receptor+ CD5- CD56+
- CD3/ γδ T Cell Receptor+ CD5- CD56-
- CD3/ γδ T Cell Receptor+ CD5- CD69+
- CD3/ γδ T Cell Receptor+ CD5- CD25+

APPROVED
AUG 13 2021
ARGUS IRB, INC.

4 References

[1] Jensen GS, Redman KA, Benson KF, Carter SG, Mitzner MA, Reeves S, Robinson L. Antioxidant bioavailability and rapid immune-modulating effects after consumption of a single acute dose of a high-metabolite yeast immunogen: results of a placebo-controlled double-blinded crossover pilot study. J Med Food. 2011 Sep;14(9):1002-10.

[2] Jensen GS, Patel D, Benson KF. A novel extract from bovine colostrum whey supports innate immune functions. II. Rapid changes in cellular immune function in humans. Prev Med. 2012 May;54 Suppl:S124-9.

[3] Jensen GS, Hart AN, Zaske LA, Drapeau C, Gupta N, Schaeffer DJ, Cruickshank JA. Mobilization of human CD34+ CD133+ and CD34+ CD133(-) stem cells in vivo by consumption of an extract from Aphanizomenon flos-aquae--related to modulation of CXCR4 expression by an L-selectin ligand? Cardiovasc Revasc Med. 2007 Jul-Sep;8(3):189-202.
